# Research on the spatial–temporal evolution of healthcare resource allocation efficiency in China

**DOI:** 10.3389/fpubh.2025.1729223

**Published:** 2026-01-15

**Authors:** Lin Wan, Jiayi Ye, Wuxiang Shi, Youhao Huang, Shangyuhui Huang

**Affiliations:** School of Humanities and Management, Guilin Medical University, Guilin, China

**Keywords:** allocation efficiency, data envelopment analysis, evaluation, healthcare resources, Malmquist index, policy

## Abstract

**Objective:**

To analyze the allocation efficiency of healthcare resources in 31 provinces, municipalities and autonomous regions in China from 2014 to 2023, and propose improvements to enhance the level of healthcare services in China.

**Methods:**

The DEA-BCC model was used to measure the static efficiency, and the Malmquist index was used to analyze the dynamic changes of total factor productivity, and the regional efficiency comparison and spatial–temporal evolution analysis were carried out.

**Results:**

The input of healthcare resources in China continued to increase, but the output was lagging behind. In 2023, the national average comprehensive efficiency was 0.869, and more than half of the provinces were DEA-ineffective. Regional differences are obvious, the efficiency of the eastern region is leading, the central region is low, and the western region fluctuates greatly. From 2014 to 2023, the average national total factor productivity was 0.984, and the efficiency showed a slight downward trend. Further decomposition finds that the lag of technological progress is a key factor restricting the improvement of total factor productivity.

**Conclusion:**

A customized resource rebalancing strategy at the provincial level should be implemented to reduce the over-allocation of hospitals and beds. Concurrently, accelerating the diffusion of medical technologies to primary care is essential, and make up for the shortcomings of technological progress through regional cooperation platforms and mandatory equipment update cycles. Furthermore, benchmarking management should be adopted, drawing on the practical experience of high-efficiency provinces in integrated healthcare alliances and digital health integration.

## 1 Introduction

The rational allocation of healthcare resources is the core foundation for safeguarding public health and advancing the high-quality development of health sector. Since the release of the “Healthy China 2030 “Blueprint (hereinafter referred to as the “Outline “) in 2016, The strategic imperative to “achieve a life expectancy of 79 years” has imposed rigorous demands for the accuracy and efficiency of medical and health resource allocation (1). Since then, a series of pivotal policies, including the “Implementation Plan for High-Quality and Efficient Healthcare Service System Construction in the 14th Five-Year Plan Period” and “the Guidelines on Further Improving the Healthcare Service System” (hereinafter referred to as the “Plan” and “Guidelines”), have been successively introduced. These documents have further underscored the objective of promoting the expansion and equitable distribution of high-quality resources and enhancing the balance of resource allocation (2–4). However, the acceleration of population aging has heightened the demand for chronic disease management, while regional economic disparities have led to uneven resource allocation and inadequate primary healthcare capacity (5, 6). These issues have collectively exacerbated the disparities in the distribution of healthcare resources across provinces, emerging as a critical bottleneck hindering the comprehensive advancement of the Healthy China strategy. Without effective improvement, this will directly impact whether the public can access high-quality healthcare services equitably and conveniently, thereby affecting societal health, well-being, and sustainable development. Consequently, scientifically evaluating the efficiency of healthcare resource allocation across provinces and identifying key areas of inefficiency has become a crucial prerequisite for optimizing resource distribution and enhancing service accessibility.

Reviewing relevant domestic scholarly research reveals studies that focus on specific provinces, as well as those adopting an urban–rural perspective. These studies highlight the polarization in the allocation of healthcare resources between urban and rural areas, emphasizing the need to increase support for rural regions. They advocate for establishing mechanisms to complement and share medical resources across urban and rural areas, while also strengthening the development of talent pools in rural areas (7–9). However, this method mainly analyzes whether the allocation of medical and health resources is effective from a static perspective, and has certain limitations in analyzing the spatial and temporal dynamic changes of resource allocation efficiency. To bridge this gap, this study comprehensively employs the BCC and Malmquist index models within Data Envelopment Analysis to systematically analyze the spatiotemporal characteristics of healthcare resource allocation efficiency in China’s 31 provinces from 2014 to 2023. It provides empirical to optimize resource allocation and enhance healthcare service efficiency.

The study makes three primary contributions. First, by integrating static (BCC) and dynamic (Malmquist) DEA models, it provides a dual perspective that reveals both the cross-sectional disparities and the temporal dynamics of efficiency changes. Second, it offers a decade-long (2014–2023), nationwide comparative analysis of all provincial-level units, which is more comprehensive in both temporal and geographical scope than studies limited to single years or specific regions. Third, it elucidates distinct spatiotemporal patterns among China’s eastern, central, and western regions, thereby furnishing policy-relevant evidence for differentiated regional governance and targeted interventions.

## 2 Materials and methods

### 2.1 Sources of materials

This study utilizes official provincial-level annual data from the National Bureau of Statistics. The dataset covers the period from 2014 to 2023 and has been collated and validated for consistency. Following the classification standards of the National Bureau of Statistics, the 31 provinces of mainland China (excluding Hong Kong, Macao, and Taiwan) are categorized into three regions: Eastern, Central, and Western. The eastern region comprises 11 provinces: Beijing, Tianjin, Hebei, Shanghai, Jiangsu, Zhejiang, Fujian, Shandong, Guangdong, Hainan, Liaoning; The Central Region encompasses 8 provinces: Shanxi, Anhui, Jiangxi, Henan, Hubei, Hunan, Jilin, Heilongjiang; The Western Region includes 12 provinces: Inner Mongolia, Guangxi, Chongqing, Sichuan, Guizhou, Yunnan, Tibet, Shaanxi, Gansu, Qinghai, Ningxia, Xinjiang.

### 2.2 Research methods

This study applies the BCC model in Data Envelopment Analysis (DEA) to conduct a static efficiency measurement, and adopts the Malmquist index to evaluate the dynamics of total factor productivity (TFP). For comparative analysis, the 31 provinces in China are categorized into three broad geographical regions—eastern, central, and western—to further elucidate the spatial distribution patterns and interregional disparities in the efficiency of healthcare resource allocation.

Data Envelopment Analysis (DEA) is a non-parametric method used to evaluate the relative efficiency of decision-making units (DMU) with multiple inputs and outputs, and it has been widely applied in healthcare efficiency measurement (10). Among various DEA models, the Charnes-Cooper-Rhodes (CCR) and Banker-Charnes-Cooper (BCC) models are the most commonly applied (11). This study utilizes the DEA-BCC model, which assumes variable returns to scale, to assess three key efficiency metrics: Comprehensive Technical Efficiency, Pure Technical Efficiency, and Scale Efficiency. Comprehensive Technical Efficiency is the product of PTE and SE, with scores ranging from 0 to 1. A score of 1 indicates relative efficiency, whereas a score below 1 indicates relative inefficiency.

The Malmquist index model is a dynamic analysis framework used to examine changes in total factor productivity across different periods for decision-making units (12). An index value greater than 1 indicates an improvement in efficiency, while a value less than 1 signifies a decline. This index can be decomposed into two components: technical efficiency change and technological progress change. Through this decomposition structure, it is possible not only to identify trends in efficiency changes but also to pinpoint the primary sources of efficiency variation.

While the DEA-BCC model provides a static evaluation of resource allocation efficiency within a single year, the Malmquist index captures efficiency changes over time and distinguishes whether they stem from improvements in technical efficiency or advancements in technology. Since static analysis alone provides a limited view of dynamic changes, combining DEA with TFP analysis enables a more holistic assessment of both the current state and long-term trends in medical resource allocation. Therefore, TFP analysis is not separate from efficiency research but rather extends it by uncovering the underlying drivers of efficiency change.

### 2.3 Selection of indicators

This study not only follows the basic requirements of the data envelopment analysis model for the number of decision making units when selecting indicators, that is, the number of decision making units is at least twice the total number of input and output indicators, but also draws on the relevant research results of Xu Pingping (13), Duan Guimin (14) and Cui Chengsen (15). Finally, the input and output indicators is determined: the input indicators selects health technicians, institutional beds and medical facilities from the three levels of manpower, material resources and institutional scale, which correspond to the core manpower, material resources and infrastructure resources of the medical and health system, and can directly reflect the accessibility of medical and health service capabilities. The output indicators focus on the core achievements of medical services, and select the number of patient visits and hospital discharges. It not only captures the intensity of service utilization, but also is a classic indicator in the field of medical and health efficiency evaluation. Together, it comprehensively reflects the supply capacity and actual service output level of the provincial medical and health system.

## 3 Results

### 3.1 Input and output indicators for healthcare resources in China, 2014–2023

The period 2014–2023 witnessed commensurate expansion across all three input indicators within China’s healthcare system. Health technicians demonstrated the most pronounced growth trajectory at an average annual rate of 5.69%, followed by institutional beds (4.92%) and medical facilities (4.48%). In contrast, the growth of output indicators remains modest and exhibits considerable volatility. The average annual growth rates for outpatient visits and hospital discharges are 2.57 and 4.45%, respectively. Notably, service output experienced significant fluctuations in 2020 due to the COVID-19 pandemic, with the number of patient visits and discharges declining by 11.23 and 13.29%, respectively. This sharp contraction can be primarily attributed to constrained access to healthcare facilities, the deferral of non-urgent medical care, and a reduced propensity of patients to seek treatment amidst pandemic control measures. A gradual recovery commenced in 2021–2022, culminating in a rebound to record-high levels by 2023. Over the ten-year span, the cumulative growth in resource inputs (health technicians: +64.54%; hospital beds: +54.12%; medical facilities: +48.32%) generally surpassed the growth in service volume (patient visits: +25.64%; hospital discharges: +47.93%). This suggests a suboptimal translation of resources into effective service delivery, highlighting issues of low utilization efficiency (Table 1).

**Table 1:** Input and output indicators for healthcare resources in China (2014–2023).

Year	Input indicators			Output indicators	
	Health technicians (10,000 persons)	Institutional beds (10,000 units)	Medical facilities (unit)	Patient visits (100 million person - times)	Hospital discharges (10,000 persons)
2014	757.98	660.12	25,860	76.04	20365.74
2015	799.74	701.52	27,587	76.99	20954.90
2016	844.46	741.06	29,140	79.30	22603.65
2017	897.83	794.01	31,056	81.81	24315.71
2018	951.95	840.37	33,009	83.05	25384.65
2019	1014.39	880.70	34,354	87.19	26502.68
2020	1066.79	910.11	35,394	77.41	22980.50
2021	1123.44	945.01	36,570	84.74	24642.10
2022	1165.80	975.00	36,976	84.16	24484.74
2023	1248.84	1017.36	38,355	95.50	30126.26

### 3.2 Analysis of DEA results on healthcare resource allocation efficiency

#### 3.2.1 Overall characteristics and temporal variation in healthcare resource allocation efficiency

An analysis of healthcare resource allocation efficiency for China’s 31 provinces in 2023 was conducted through the DEA-BCC model employing DEAP 2.1 software, from which the evaluation results were obtained. Scale efficiency reflects the degree of alignment between the scale of healthcare resource inputs and outputs, while pure technical efficiency reflects the utilization effectiveness determined by management and technology application levels (Table 2).

**Table 2:** Efficiency of healthcare resource allocation in 31 provinces of China in 2023.

Region	Comprehensive technical efficiency	Pure technical efficiency	Scale efficiency	Returns to scale	DEA validity
Beijing	1.000	1.000	1.000	-	Valid
Tianjin	0.903	0.993	0.909	irs	Invalid
Hebei	0.801	0.801	1.000	-	Invalid
Shanghai	1.000	1.000	1.000	-	Valid
Jiangsu	0.902	0.910	0.991	drs	Invalid
Zhejiang	1.000	1.000	1.000	-	Valid
Fujian	0.885	0.885	1.000	-	Invalid
Shandong	0.943	1.000	0.943	drs	Weakly efficient
Guangdong	1.000	1.000	1.000	-	Valid
Hainan	0.696	0.936	0.743	irs	Invalid
Liaoning	0.708	0.711	0.996	irs	Invalid
Eastern region	0.894	0.931	0.962		
Shanxi	0.637	0.646	0.986	irs	Invalid
Anhui	0.846	0.853	0.992	drs	Invalid
Jiangxi	0.870	0.870	1.000	-	Invalid
Henan	0.924	1.000	0.924	drs	Weakly efficient
Hubei	1.000	1.000	1.000	-	Valid
Hunan	0.916	0.923	0.992	drs	Invalid
Jilin	0.644	0.659	0.976	irs	Invalid
Heilongjiang	0.816	0.826	0.988	irs	Invalid
Central region	0.832	0.847	0.982		
Inner Mongolia	0.662	0.672	0.986	irs	Invalid
Guangxi	1.000	1.000	1.000	-	Valid
Chongqing	1.000	1.000	1.000	-	Valid
Sichuan	0.999	1.000	0.999	drs	Weakly efficient
Guizhou	0.953	0.957	0.995	drs	Invalid
Yunnan	0.947	0.953	0.994	drs	Invalid
Tibet	0.521	1.000	0.521	irs	Weakly efficient
Shaanxi	0.853	0.855	0.998	drs	Invalid
Gansu	0.864	0.889	0.971	irs	Invalid
Qinghai	0.724	0.969	0.747	irs	Invalid
Ningxia	0.935	1.000	0.935	irs	Weakly efficient
Xinjiang	1.000	1.000	1.000	-	Valid
Western region	0.872	0.941	0.929		
Mean	0.869	0.913	0.954		

The DEA efficiency evaluation revealed a distinct three-tier distribution among the 31 regions. Eight regions—Beijing, Shanghai, Zhejiang, Guangdong, Hubei, Guangxi, Chongqing, and Xinjiang—were identified as DEA-efficient, accounting for 25.8% of the total. Five regions—Shandong, Henan, Sichuan, Tibet, and Ningxia—demonstrated weak DEA efficiency, characterized by pure technical efficiency scores of 1 but overall technical efficiency scores below 1, representing 16.1% of cases. The remaining 18 regions, including Tianjin, Hebei, and Jiangsu, were classified as DEA-ineffective with overall technical efficiency scores below 1, constituting 58.1% of the total.

At the national level, the average comprehensive technical efficiency across China’s 31 provinces was 0.869, with 12 regions falling below this mean. The lowest efficiency was observed in Tibet (0.521), followed by Shanxi (0.637). The average pure technical efficiency stood at 0.913, yet 12 regions remained below the average. With Shanxi (0.646) and Jilin (0.659) recording the lowest values. Scale efficiency reached a relatively high mean of 0.954, with 7 regions performing below this level—Tibet (0.521) and Hainan (0.743) being the least efficient. Regarding returns to scale, 11 regions including Tianjin and Hainan, exhibited increasing returns, nine regions, such as Jiangsu and Shandong showed decreasing returns, the remaining 11 regions maintained constant returns to scale. Geographically, the eastern region achieved the highest comprehensive efficiency, followed by the western and central regions. The eastern region’s strong performance stems from its high pure technical efficiency and favorable scale efficiency. The western region’s greatest advantage lies in its leading pure technical efficiency, though its scale efficiency remains relatively low. In contrast, the central region’s inefficiency was primarily attributable to significantly lower pure technical efficiency, while near-optimal scale efficiency. This indicates the issue lies not in scale size but in how to utilize existing inputs more effectively.

Applying the same methodology to analyze healthcare resources across China’s 31 provinces from 2014 to 2023 reveals stagnant growth in national average comprehensive efficiency, which increased marginally from 0.868 to 0.869, remaining suboptimal. Seven regions—Shanghai, Zhejiang, Henan, Tibet, Guangdong, Guangxi, and Sichuan—maintained efficiency throughout the decade. Chongqing achieved efficiency in nine out of ten years, except for 2018. Hubei experienced a low level of healthcare resource allocation efficiency in 2020 due to pandemic-induced resource constraints, but achieved efficiency in the remaining nine years. Four regions—Shandong, Jiangxi, Hunan, and Ningxia—exhibited a volatile trajectory, frequently alternating between relative efficiency and inefficiency over the decade. Meanwhile, eighteen regions, including Tianjin, Hebei, Jiangsu, and Fujian, demonstrated persistent inefficiency in most years, with minor fluctuations in efficiency scores.

#### 3.2.2 Analysis of input redundancy and output deficiency in DEA-ineffective provinces

To further identify the sources of inefficiency, a slack variable analysis was conducted on provinces with low DEA efficiency. The results indicate that inefficiency is primarily attributable to input excess rather than output shortfall, as detailed in Tables 3, 4.

**Table 3:** Input redundancy of healthcare resources in DEA-inefficient regions.

	Relaxation variable S-				Input redundancy rate		
Region	Medical facilities (unit)	Institutional beds (10,000 units)	Health technicians (10,000 persons)	Summary	Medical facilities (unit)	Institutional beds (10,000 units)	Health Technicians (10,000 persons)
Tianjin	152.448	0.000	1.482	153.929	0.333	0.000	0.111
Hebei	561.342	3.285	0.000	564.627	0.226	0.062	0.000
Jiangsu	72.823	0.000	0.000	72.823	0.034	0.000	0.000
Fujian	0.000	0.000	0.000	0.000	0.000	0.000	0.000
Hainan	0.000	0.000	0.015	0.015	0.000	0.000	0.002
Liaoning	272.115	0.000	0.000	272.115	0.177	0.000	0.000
Shanxi	192.038	0.000	0.000	192.038	0.139	0.000	0.000
Anhui	0.000	1.927	0.000	1.927	0.000	0.042	0.000
Jiangxi	9.918	0.779	0.000	10.697	0.009	0.023	0.000
Hunan	0.000	0.078	0.000	0.078	0.000	0.001	0.000
Jilin	13.395	0.000	0.000	13.395	0.015	0.000	0.000
Heilongjiang	327.349	1.789	0.000	329.138	0.262	0.065	0.000
Inner Mongolia	22.979	0.000	0.492	23.471	0.027	0.000	0.021
Guizhou	227.119	0.000	0.000	227.119	0.147	0.000	0.000
Yunnan	0.000	0.000	0.000	0.000	0.000	0.000	0.000
Shaanxi	0.000	0.000	1.133	1.133	0.000	0.000	0.029
Gansu	0.000	0.000	0.000	0.000	0.000	0.000	0.000
Qinghai	15.233	0.000	0.055	15.288	0.063	0.000	0.010

**Table 4:** Output insufficiency of healthcare resources in DEA-inefficient regions.

	Relaxation variable S+			Output insufficient rate	
Region	Patient visits (100 million person - times)	Hospital discharges (10,000 persons)	Summary	Patient visits (100 million person - times)	Hospital discharges (10,000 persons)
Tianjin	0.000	0.000	0.000	0.000	0.000
Hebei	0.000	0.000	0.000	0.000	0.000
Jiangsu	0.000	0.000	0.000	0.000	0.000
Fujian	0.000	0.000	0.000	0.000	0.000
Hainan	0.000	0.000	0.000	0.000	0.000
Liaoning	0.038	0.000	0.038	0.020	0.000
Shanxi	0.000	0.000	0.000	0.000	0.000
Anhui	0.000	0.000	0.000	0.000	0.000
Jiangxi	0.000	0.000	0.000	0.000	0.000
Hunan	0.046	0.000	0.046	0.011	0.000
Jilin	0.000	0.000	0.000	0.000	0.000
Heilongjiang	0.525	0.000	0.525	0.437	0.000
Inner Mongolia	0.000	0.000	0.000	0.000	0.000
Guizhou	0.272	0.000	0.272	0.130	0.000
Yunnan	0.000	0.000	0.000	0.000	0.000
Shaanxi	0.000	0.000	0.000	0.000	0.000
Gansu	0.241	0.000	0.241	0.199	0.000
Qinghai	0.000	0.000	0.000	0.000	0.000

From the perspective of input redundancy, an excess number of hospitals constitutes the most prominent issue among DEA-inefficient provinces. Significant hospital redundancy is observed in Hebei, Heilongjiang, Liaoning, Guizhou, and Tianjin. Among these, Tianjin exhibits the highest redundancy rate at 33.3%, followed by Heilongjiang (26.2%) and Hebei (22.6%). This suggests that investment in medical facilities in these provinces has surpassed current service demand, pointing to issues of institutional over-expansion and functional overlap. In contrast, redundancy in beds and health technicians is more concentrated in a limited set of provinces. Bed redundancy rates are notably high in Hebei (6.2%) and Heilongjiang (6.5%). Similarly, health technician redundancy rates are elevated in Inner Mongolia (2.1%) and Shaanxi (2.9%). This pattern reflects a regional mismatch between resource allocation and service demand, rather than a nationwide oversupply.

Regarding output shortfalls, 18 DEA-inefficient provinces showed no deficiency in hospital discharges. Only a few provinces exhibited significant shortfalls in outpatient and emergency visits. Heilongjiang had the highest deficiency rate at 43.7%, followed by Gansu (19.9%) and Guizhou (13%). This indicates that low efficiency stems primarily from underutilization and misallocation of healthcare investments, not from a lack of service demand. Notably, several DEA-inefficient provinces exhibit negligible slack values. This indicates that their operations are close to the production frontier and require optimization through technological or managerial upgrades, rather than resource cutbacks.

#### 3.2.3 Regional disparities in healthcare resource allocation efficiency

**Figure 1:**
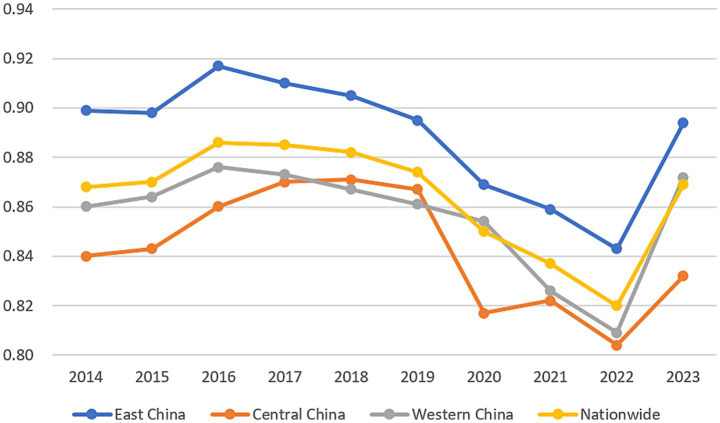
Comprehensive technical efficiency of healthcare resource allocation in 31 provinces (2014–2023).

By dividing China’s 31 provinces into eastern, central, and western regions, it was found that there are significant regional disparities in the efficiency of healthcare resource allocation across the country. Overall, the eastern region consistently demonstrated the highest comprehensive efficiency, followed by the western region, while the central region exhibited the lowest efficiency. When compared to the national average comprehensive efficiency, the eastern region persistently surpassed this benchmark. The western region slightly exceeded the national average in 2020 and 2023 but remained below it during the other eight years, while the central region consistently performed below average throughout the entire period. Furthermore, the eastern region maintained relatively high comprehensive technical efficiency over most years, demonstrating its advantage in healthcare resource allocation. Although the western region displayed lower efficiency levels, it showed gradual improvement. Conversely, the central region experienced significant fluctuations in comprehensive efficiency, with an overall downward trend (Figure 1).

**Figure 2:**
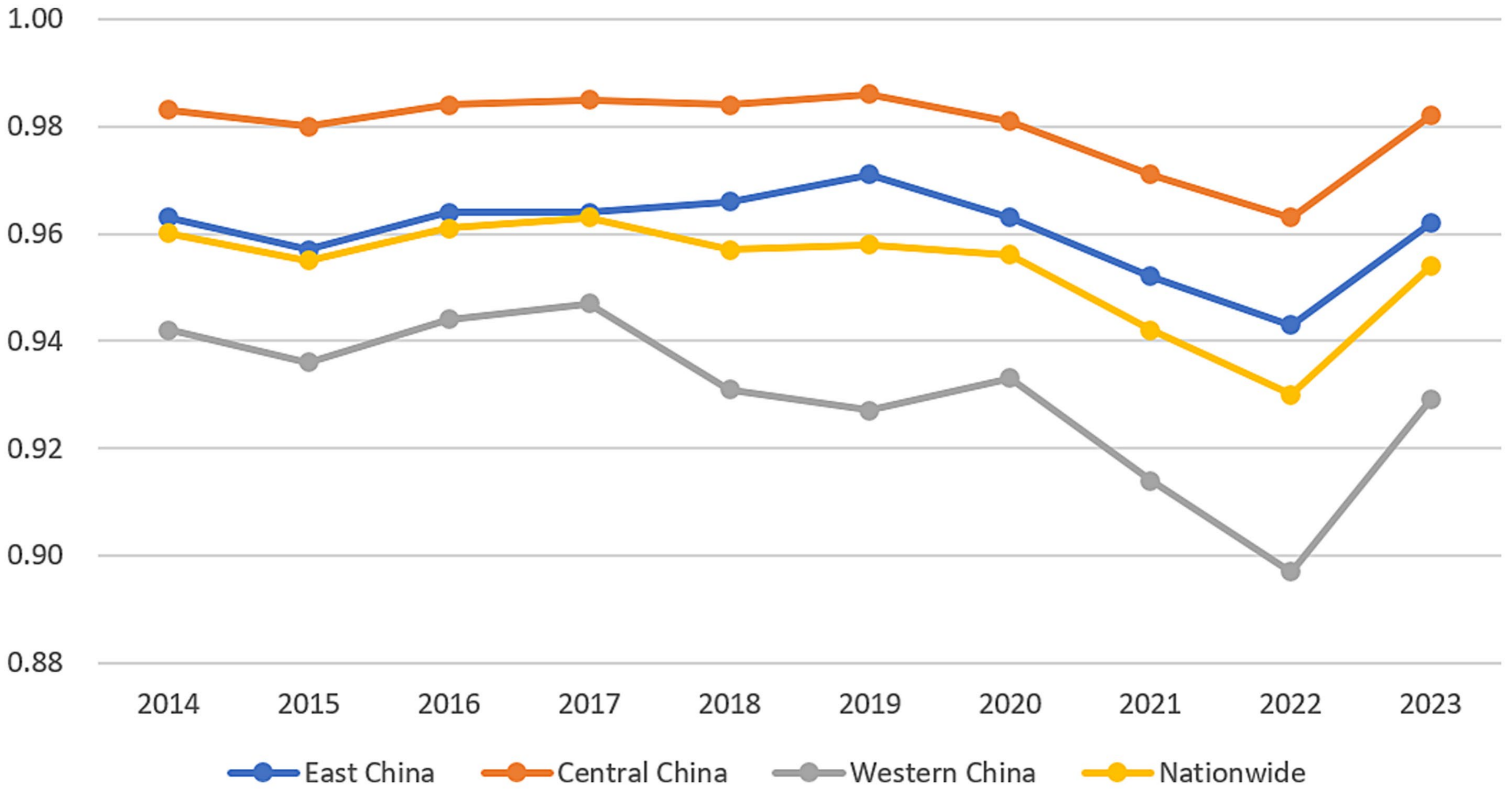
Scale efficiency of healthcare resource allocation in 31 provinces (2014–2023).

**Figure 3:**
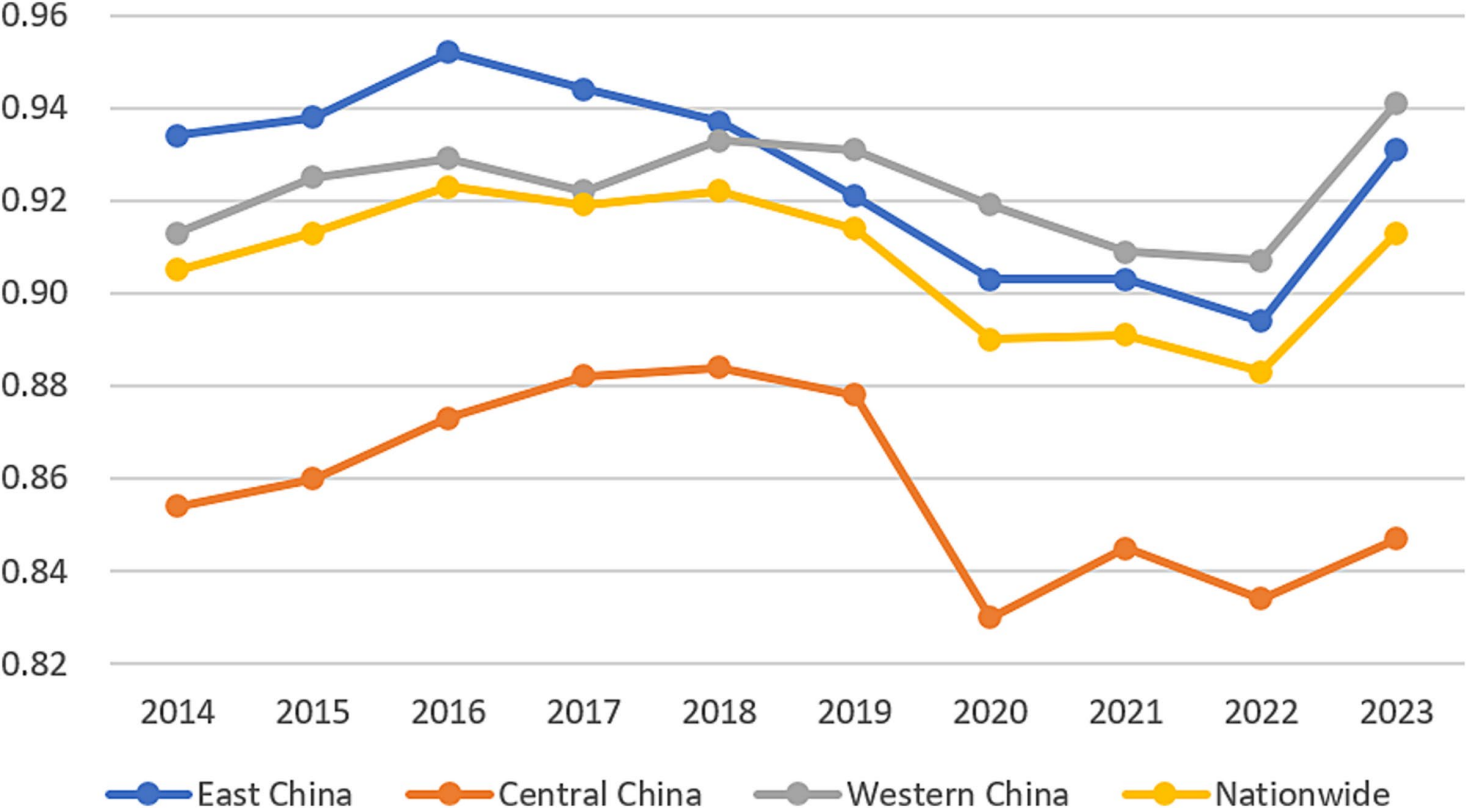
Pure technical efficiency of healthcare resource allocation in 31 provinces (2014–2023).

Further decomposition revealed that distinct regional patterns in both scale and pure technical efficiency between the eastern, central, and western regions. Regarding scale efficiency, the eastern and central regions exhibit fluctuations at relatively high levels but show an overall stabilizing trend, indicating reasonably optimized resource allocation scales. In contrast, the western region experienced greater volatility, displaying a general decline before 2022 followed by moderate improvement in 2023, yet remaining consistently below the national average (Figure 2). In the terms pure technical efficiency, the eastern region maintained a leading position despite a continuous decline since its peak in 2016. The central region exhibited considerable fluctuation at levels substantially below the national average, while the western region showed an oscillating yet upward trajectory (Figure 3). Comprehensively, the eastern region sustained superior performance across efficiency dimensions, reflecting its advanced overall efficiency in healthcare resource allocation. Although the central and western regions started from lower baselines in certain dimensions, their gradual improvement signals potential for future development. The national trend closely mirrors that of the eastern region, suggesting that efficiency gains in healthcare resource allocation nationwide are largely driven by the eastern regions performance.

### 3.3 Spatiotemporal evolution of total factor productivity in healthcare resources

#### 3.3.1 Temporal dynamics of total factor productivity and its decomposition in healthcare resources

Building upon the initial analysis, the Malmquist model was implemented through DEAP 2.1 software to evaluate input–output indicators of healthcare resources across China’s 31 provinces from 2014 to 2023. This yielded the total factor productivity index for healthcare resource allocation efficiency in China, along with its decomposition results. Within this framework, technical efficiency reflects the degree of comprehensiveness in utilizing healthcare resources, while technical progress manifests as enhancements in healthcare personnel capabilities or advancements in medical technology (Table 5).

**Table 5:** Changes in total factor productivity index of healthcare resources (2014–2023).

Year	Technical efficiency	Technological progress	Pure technical efficiency	Scale efficiency	Total factor productivity
2014—2015	1.003	0.964	1.009	0.994	0.967
2015—2016	1.020	0.988	1.013	1.007	1.007
2016—2017	1.000	0.994	0.998	1.002	0.994
2017—2018	0.996	0.983	1.003	0.992	0.979
2018—2019	0.989	0.993	0.990	0.999	0.983
2019—2020	0.968	0.855	0.968	1.000	0.827
2020—2021	0.984	1.043	1.001	0.982	1.026
2021—2022	0.976	0.971	0.991	0.985	0.948
2022—2023	1.068	1.082	1.040	1.027	1.156
Mean	1.000	0.984	1.001	0.999	0.984

**Figure 4:**
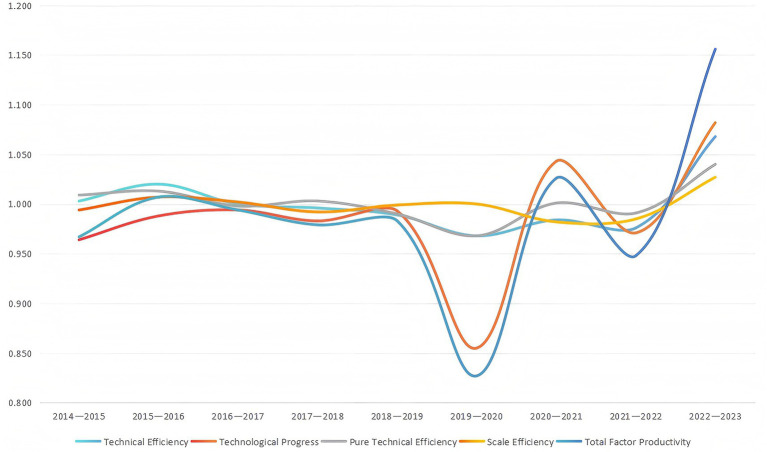
Annual trends in total factor productivity and its decomposition of healthcare resources in China (2014–2023).

From the perspective of time evolution, the average total factor productivity of national medical and health resources from 2014 to 2023 is 0.984, that is, the average annual decline in the efficiency of national medical and health resources is 1.6%. However, this overall slight decline is not evenly distributed over time, but is mainly driven by sharp fluctuations in a particular year, as shown in Figure 4. TFP shows the characteristics of “stable first, then falling, and then rising rapidly.” Its fluctuation can be attributed to two key periods: one is the “efficiency trough” (TFP = 0.827) caused by the impact of COVID-19 from 2019 to 2020, and the other is the “strong rebound “(TFP = 1.156) accompanied by service recovery and technology application from 2022 to 2023. If the abnormal value of 2019–2020 is excluded, the mean value of TFP in the remaining period is 1.008, indicating that it has the ability to be basically stable and slightly improved under normal conditions. Further decomposition shows that the average annual increase of pure technical efficiency is 0.1%, and the average annual decrease of scale efficiency is 0.1%, and the fluctuation is relatively flat. The average annual decline of 1.6% in technological progress has become the main reason for the decline in total factor productivity, that is, the lag of medical and health technology and the slow renewal of equipment have seriously hindered the improvement of total factor productivity. It can be seen that making up for the lag of technological progress is the key focus to improve the total factor productivity of national healthcare resources.

Specifically, from 2014 to 2019, the total factor productivity basically fluctuated around 1, and the decline was relatively limited. The main constraints came from the continuous weakness of the technological progress index, while the overall technical efficiency and scale efficiency remained relatively stable, indicating that the management level of medical resource allocation was relatively stable, but the pace of technological innovation and renewal was relatively lagging behind. From 2019 to 2020, TFP fell to the lowest value of 0.827 in the whole period, showing a sharp decline. The decomposition results show that the decline is mainly caused by the significant decline in the technical progress index (0.855) and technical efficiency (0.968), while the scale efficiency remain basically stable, indicating that the efficiency loss in this stage is mainly due to the exogenous impact of the COVID-19 epidemic on the supply and application of conventional medical services, rather than the structural imbalance of resource allocation. In the post-epidemic period, TFP showed obvious recovery growth. In 2020–2021, TFP rebounded to 1.026, and in 2022–2023, it further jumped to 1.156, and technical efficiency, technological progress and scale efficiency improved simultaneously, reflecting that China’s medical system showed strong system resilience and adjustment ability under the joint action of factors such as centralized demand release and accelerated application of technology.

Further decomposition found that technical efficiency remained negative in most intervals except 2014–2017 and 2022–2023, yet demonstrated overall stability. However, pure technical efficiency and scale efficiency exhibited divergent trends during the same period: pure technical efficiency increased by 0.9% in 2014–2015 while scale efficiency decreased by 0.6%; in 2016–2017, scale efficiency increased by 0.2% as pure technical efficiency fell by 0.2%; both pure technical efficiency and scale efficiency showed positive growth in 2015–2016 and 2022–2023. Overall, pure technical efficiency generally trended upward but exhibited relative volatility. Annual changes from 2014 to 2023 were 0.9, 1.3, −0.2%, 0.3, −1%, −3.2, 0.1, −0.9%, and 4%, respectively, demonstrating pronounced fluctuations. While scale efficiency, though mostly negative, exhibited milder fluctuations of −0.6, 0.7, 0.2, −0.8%, −0.1, 0.0, −1.8%, −1.5, and 2.7%, indicating relative stability. Meanwhile, technological progress experienced declines during 2014–2020 and 2021–2022, with decreases of 3.6, 1.7, 14.5, and 2.9% during 2014–2015, 2017–2018, 2019–2020, and 2021–2022, respectively. It did not show positive growth until 2020–2021 and 2022–2023, increasing by 4.3 and 8.2%, respectively.

#### 3.3.2 Interprovincial disparities in total factor productivity of healthcare resources

From the perspective of interprovincial total factor productivity, an overall decline was observed across China’s 31 provinces. Only four provinces—Beijing, Heilongjiang, Shaanxi, and Ningxia—achieved marginal TFP improvements, while the remaining 27 provinces experienced deterioration. Tibet saw the sharpest efficiency drop, followed sequentially by Hebei, Jiangxi, Anhui, Hunan, and Shanghai. Regional analysis revealed the severity of TFP decline followed the order: central > eastern > western regions. Decomposition analysis at the provincial level showed that 11 provinces (Hebei, Hainan, Anhui, Jiangxi, Henan, Hunan, Jilin, Guizhou, Yunnan, Tibet, Qinghai) exhibited declines in technical efficiency, whereas the other 20 maintained technical efficiency values ≥1. Among these 31 provinces, only Jiangsu and Fujian demonstrated improved technical efficiency despite decreased pure technical efficiency. Conversely, among the 11 provinces with declining overall technical efficiency, Hainan, Henan, Tibet, and Qinghai all experienced increases in pure technical efficiency. The remaining provinces displayed consistent directional movements between technical and pure technical efficiency. Furthermore, the technical progress values remained below 1 across all provinces, reaffirming its critical role in driving TFP growth (Table 6).

**Table 6:** Interprovincial total factor productivity of medical and healthcare resources.

Region	Technical efficiency	Technological progress	Pure technical efficiency	Scale efficiency	Total factor productivity
Beijing	1.010	0.993	1.009	1.001	1.004
Tianjin	1.001	0.990	1.005	0.995	0.991
Hebei	0.980	0.981	0.979	1.002	0.962
Shanghai	1.000	0.974	1.000	1.000	0.974
Jiangsu	1.000	0.986	0.998	1.001	0.986
Zhejiang	1.000	0.992	1.000	1.000	0.992
Fujian	1.001	0.983	0.999	1.001	0.983
Shandong	1.010	0.985	1.000	1.010	0.995
Guangdong	1.000	0.984	1.000	1.000	0.984
Hainan	0.989	0.986	1.004	0.986	0.975
Liaoning	1.000	0.985	1.000	1.000	0.985
Eastern region	0.999	0.985	0.999	1.000	0.985
Shanxi	1.011	0.985	1.011	1.001	0.996
Anhui	0.992	0.980	0.992	1.000	0.972
Jiangxi	0.985	0.980	0.985	1.000	0.965
Henan	0.997	0.981	1.000	0.997	0.978
Hubei	1.002	0.981	1.000	1.002	0.983
Hunan	0.990	0.984	0.991	0.999	0.974
Jilin	0.999	0.983	0.999	0.999	0.982
Heilongjiang	1.025	0.982	1.025	1.000	1.006
Central region	1.000	0.982	1.000	1.000	0.982
Inner Mongolia	1.010	0.984	1.008	1.002	0.993
Guangxi	1.000	0.981	1.000	1.000	0.981
Chongqing	1.000	0.985	1.000	1.000	0.985
Sichuan	1.005	0.983	1.000	1.005	0.988
Guizhou	0.995	0.985	0.995	0.999	0.979
Yunnan	0.994	0.983	0.995	0.999	0.977
Tibet	0.963	0.980	1.000	0.963	0.944
Shaanxi	1.018	0.985	1.017	1.001	1.003
Gansu	1.002	0.984	1.004	0.999	0.986
Qinghai	0.999	0.989	1.008	0.991	0.988
Ningxia	1.017	0.988	1.010	1.007	1.005
Xinjiang	1.010	0.987	1.008	1.002	0.997
Western region	1.001	0.985	1.004	0.997	0.986
Mean	1.000	0.984	1.001	0.999	0.984

**Figure 5:**
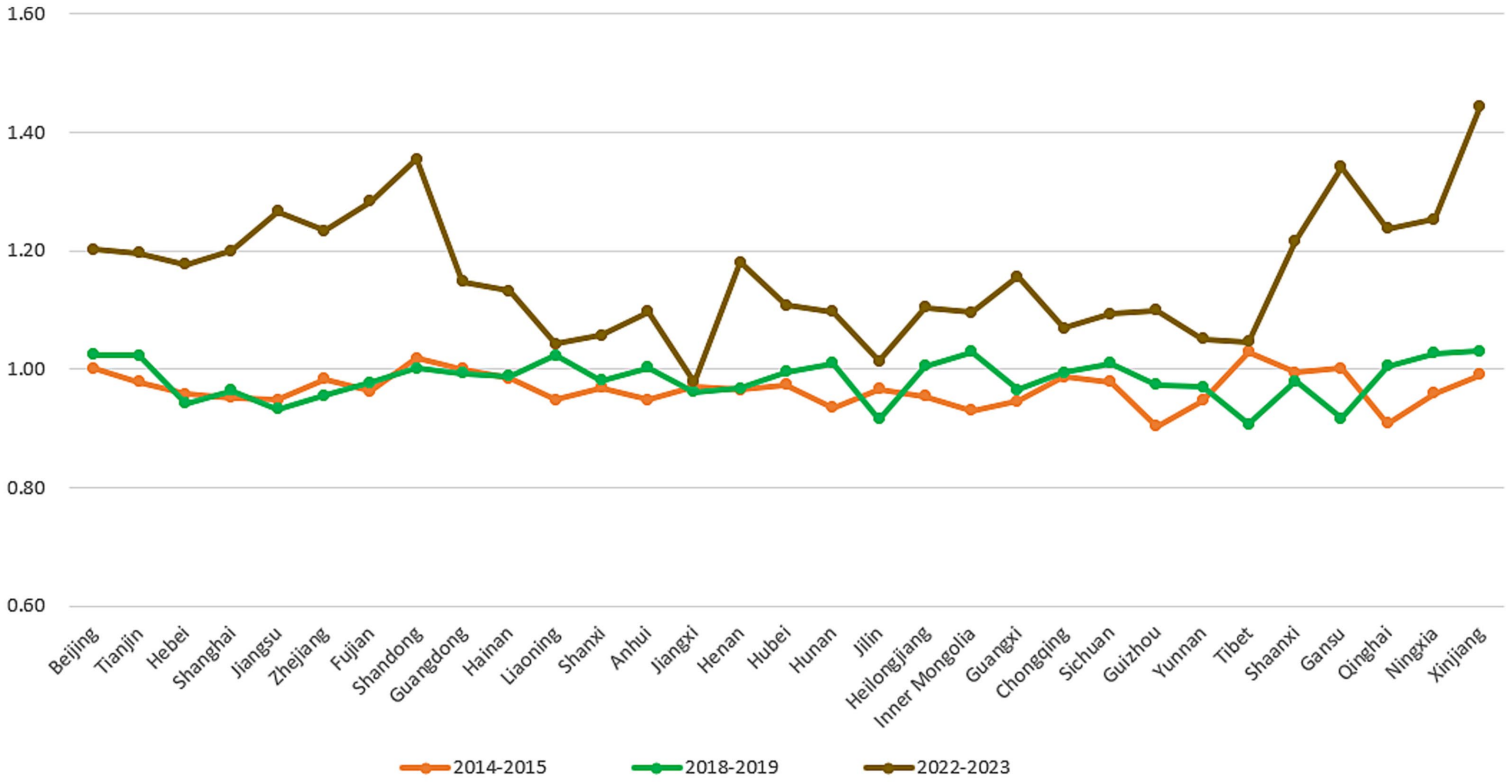
Interprovincial total factor productivity across three time periods.

To further examine the spatiotemporal evolution of interprovincial healthcare resource allocation efficiency in China from 2014 to 2023, we analyzed the total factor productivity data for each province across three time periods: 2014–2015, 2018–2019, and 2022–2023 (Figure 5). Temporally, the 2014–2015 period was characterized by generally low TFP levels nationwide. Only five provinces—Beijing, Shandong, Guangdong, Tibet, and Gansu—achieved TFP growth, while Tianjin, Zhejiang, Hainan, Chongqing, Shaanxi, and Xinjiang maintained relatively high TFP values despite remaining below 1. Conversely, remote provinces such as Hunan, Inner Mongolia, Guizhou, and Qinghai experienced pronounced efficiency declines. By 2018–2019, several provinces previously ranking in the middle-to-lower tier—notably Tianjin, Liaoning, Inner Mongolia, Sichuan, Qinghai, Ningxia, and Xinjiang—had transformed into rapidly improving regions. Provinces like Liaoning, Hunan, and Guizhou also showed marked progress. However, Jilin, Tibet, and Gansu experienced accelerated declines in TFP, emerging as new laggards. The 2022–2023 period witnessed positive TFP growth across 30 provinces except Jiangxi, with Shandong, Gansu, and Xinjiang demonstrating particularly remarkable gains. Spatially, western provinces achieved comprehensive high growth benefiting from resource upgrades and policy support. Eastern provinces advanced steadily through technological innovation and mature healthcare systems. The central region, however, showed pronounced internal disparities: provinces like Henan and Hubei joined the growth tier, while Jiangxi recorded negative growth.

### 3.4 Robustness tests

To assess the robustness and reliability of the findings, this section performs robustness checks along two dimensions: model specification and variable selection. Given that Data Envelopment Analysis is sensitive to its underlying assumptions and input–output variable sets, we re-estimate efficiency scores by altering key model configurations. This approach tests whether the core conclusions remain unchanged under alternative methodological setups.

First, in the context of static efficiency analysis, we recognize that medical service output is multidimensional and that the original indicator, “number of discharged patients”primarily captures service volume. To better reflect the technical complexity and resource intensity of care, we substitute it with “Inpatient Surgical Procedures” sourced from the National Bureau of Statistics. Efficiency scores are then re-estimated using the DEA-BCC model and compared against the baseline. The Spearman rank correlation test indicates a statistically significant positive correlation between the two sets of efficiency rankings (*ρ* = 0.66, *p* < 0.001), demonstrating that the static efficiency evaluation maintains moderate stability and robustness under this alternative output measure.

Second, for the dynamic efficiency analysis, we examine the potential influence of returns-to-scale assumptions on total factor productivity measurement. Specifically, the Malmquist index is recalculated using the DEA-CCR model and compared with the results from the DEA-BCC model. A significant positive correlation is found between the indices derived from the two models (ρ = 0.56, *p* < 0.001). Although there are some differences in the specific values of the productivity index due to different assumptions of returns to scale, the overall trend is consistent, indicating that the conclusion of this paper on the dynamic evolution of medical resource allocation efficiency has good robustness.

## 4 Discussion and policy implications

### 4.1 Suboptimal efficiency amid disproportionate input–output growth

Between 2014 and 2023, China’s healthcare system underwent substantial expansion in both infrastructure and human resources, evidenced by cumulative growth of 64.54% in health technicians, 54.12% in hospital beds, and 48.32% in healthcare institutions. However, the growth rates for outpatient visits and hospital discharges were only 25.64 and 47.93%, respectively—significantly lower than the pace of resource investment. The asymmetry between input and output growth rates indicates a structural imbalance in the allocation of medical resources. Further analysis of slack variables in DEA-inefficient provinces reveals that this imbalance is manifested as the coexistence of input excess and output shortfall. This shows that the efficiency of resource utilization has not been improved simultaneously with the input, which is reflected in the obvious resource mismatch at the provincial level. In 2023, the average comprehensive technical efficiency across 31 provinces stood at 0.869, with average pure technical efficiency and scale efficiency at 0.913 and 0.954, respectively. Over half of the regions failed to achieve DEA effectiveness, these findings collectively indicate suboptimal resource utilization efficiency nationwide, highlighting substantial potential for optimization.

Therefore, the government should comprehensively account for local healthcare demands and demographic distribution patterns, enhance regional health planning, and optimize existing medical resources. It is essential to avoid haphazard expansion of tertiary hospitals to achieve precise alignment between resource allocation and public health needs. Concurrently, policy should encourage patients with common and chronic conditions to utilize primary care facilities, thereby fostering complementary development and synergistic use of healthcare resources. Furthermore, establishing multidimensional performance metrics centered on service quality, patient satisfaction, and cost containment is essential to transition from a volume-oriented to an efficiency-oriented healthcare delivery (16). Finally, according to local conditions, redundant human resources should be transformed into talents that meet the needs of different levels of institutions through standardized training, specialist training and other ways.

### 4.2 Marked interregional variations: eastern high versus central-western low efficiency

From a regional perspective, the eastern region demonstrates the highest efficiency in healthcare resource allocation, with its comprehensive technical efficiency significantly exceeding the national average. In contrast, the central region exhibits the lowest efficiency, while the western region slightly outperforms the central region yet remains below the national benchmark. Three primary factors explain these disparities. First, the eastern region benefits from robust economic foundations within the Yangtze River Delta, Pearl River Delta, and Bohai Rim economic circles, which consistently support technological upgrades and talent development. Since the 18th National Congress of the Communist Party of China, the Party Central Committee has introduced a series of major regional strategies, including Beijing-Tianjin-Hebei coordinated development and Yangtze River Delta integration have enabled the eastern region to pioneer tightly-knit medical alliances and telemedicine networks, thereby enhancing technical efficiency and resource coordination. Second, although the central region rise strategy emphasizes equalization of public services, specific policies have largely focused on infrastructure construction, with insufficient attention to talent cultivation and technological innovation. Third, provinces including Xinjiang, Tibet, and Guizhou have utilized national counterpart assistance mechanisms to establish preliminary regional collaboration and telemedicine platforms, enhancing healthcare service capabilities in select remote areas. Additionally, regional strategies like the Chengdu-Chongqing Economic Circle have brought new opportunities to the western regions, partially alleviating healthcare workforce shortages. This dual approach of “external support and endogenous capacity building” has progressively optimized healthcare resource allocation efficiency in western China.

The Guiding Opinions call for optimized spatial distribution of healthcare resources to bridge urban–rural and interregional disparities, and narrow the gap in healthcare resource distribution between central-western and eastern regions. Therefore, Implementation should prioritize telemedicine infrastructure, medical consortium development, and targeted technical assistance to foster balanced regional development. According to the “Opinions of the Central Committee of the Communist Party of China and the State Council on Promoting High-Quality Development in the central region in the New Era” synergies should be strengthened with major regional strategies such as Beijing-Tianjin-Hebei coordination and Yangtze River Economic Belt integration. By absorbing the relocation of high-quality medical resources from the Beijing-Tianjin-Hebei region and collaborating with the Yangtze River Economic Belt to establish a cross-regional medical technology coordination system, the efficiency gap in regional healthcare resource allocation can be narrowed. Concurrently, refined incentive policies for healthcare professionals, promoting their migration to underdeveloped central and western regions through enhanced compensation and welfare measures (17).

### 4.3 Technological progress as the primary constraining factor

Between 2014 and 2023, China experienced an average annual decline of 1.6% in total factor productivity, with a mean value of 0.984. Technological efficiency remained stable during this period, while technological regression—also decreasing at 1.6% per year—emerged as the principal determinant of TFP deterioration. One contributing factor is the significant lag in the iterative upgrading of medical equipment, particularly within primary healthcare institutions at or below the county level, the allocation rate of high-end medical equipment remains low, and advanced technological innovations struggle to permeate effectively to these grassroots levels (18). Secondly, a structural shortage exists in the supply of intermediate and senior healthcare technical personnel. In particular, the central and western regions face an insufficient total number of such personnel, imperfect talent echelon development, and a lag in the professional competency of staff to operate new technologies and equipment. This makes it difficult to translate hardware investments into tangible improvements in service efficiency (19).

From a spatial evolution perspective, the eastern and western regions demonstrated gradual TFP improvement, whereas central China exhibited lower levels and slower growth. Moreover, all provinces recorded technical progress values below 1, reflecting a dual challenge of systemic inefficiency and significant regional fragmentation in healthcare technology advancement. Although the national implementation plan advocates for “expanding high-quality medical resources and achieving balanced regional distribution” (2), policy execution displays marked regional disparities. The eastern region leverages economic advantages to attract skilled professionals and introduce advanced equipment, creating a virtuous cycle; the western region has established telemedicine platforms through policy support, while in the central region, high-quality technical resources are concentrated in provincial capitals. This centralization, coupled with slow equipment renewal and staffing shortages in county-level institutions, has created a hierarchical fragmentation in technology diffusion that fundamentally constrains TFP growth.

Consequently, the Healthy China 2030 Blueprint should be operationalized by institutionalizing healthcare technology R&D investment as a performance metric in local government health assessments, while curricular expansion in medical technology innovation should be implemented at central China’s universities to cultivate an indigenous technical talent pool. Furthermore, a structured collaboration framework for technology translation should be established between eastern and central regions, supported by creating specialized intermediary hubs for medical technology transfer and regional healthcare centers. In alignment with the Implementation Plan’s primary-care competency standards, sustainable training mechanisms for grassroots medical personnel must be established. Telemedicine operations and smart device utilization should be incorporated into competency evaluations, ensuring technologies become both accessible and applicable in routine practice.

### 4.4 Characteristics and optimization enlightenment of efficiency frontier provinces

The replicable common characteristics observed in the eight DEA-efficient provinces identified in this study offer a clear optimization pathway for inefficient provinces. First, they exhibit a coordinated resource input structure that effectively avoids single-factor redundancy. These provinces maintain a more balanced ratio among health technicians, beds, and institutions (20). In contrast, inefficient provinces frequently exhibit problems of over-investment in a single resource, exemplified by the excessive number of hospitals in Hebei and the surplus of beds in Heilongjiang. This observed redundancy underscores that an optimal allocation structure is more critical than merely increasing investment. Second, technology and management innovation drive intensive, quality-oriented development. Most of these efficient provinces are pioneers in healthcare reform. Regions such as the Yangtze River Delta and the Pearl River Delta lead in promoting smart hospitals and telemedicine to enhance service efficiency (21). Meanwhile, Guangxi and Chongqing have facilitated resource allocation to grassroots levels through the establishment of tight-knit medical alliances (22). Inefficient provinces, conversely, commonly lag in technology adoption and management practices. Third, they possess sound regional coordination mechanisms. Leveraging national-level strategies, they have constructed cross-regional cooperation networks, breaking down the localization of resources through a dual approach of “external linkage and internal integration” (23). The Beijing-Tianjin-Hebei coordinated development initiative and the Yangtze River Delta integration strategy serve as typical examples.

To enhance the resource allocation efficiency of inefficient provinces, it is imperative to move beyond the mindset of merely increasing investment. In accordance with “Guidance on Optimizing the Layout and Construction of Primary Healthcare Institutions” (24), a dynamic resource monitoring and adjustment mechanism should be established. This involves regularly evaluating and rebalancing key factors such as beds and institutions, and implementing a two-way flow mechanism between tertiary and primary healthcare—combining downward rotations with dispatched training. This approach not only revitalizes redundant resources but also strengthens primary-level service capabilities.

It is equally crucial to strengthen the dual drivers of technology and management. On one hand, it is essential to promote state-advocated digital service models, such as the “telemedicine service network” and the model of “distributed inspection with centralized diagnosis.” On the other hand, the requirements of the DRG/DIP payment reform must be fully implemented to promote the integration of digital tools with performance management and regulatory mechanisms.

Furthermore, based on “the Opinions on Further Ensuring and Improving People’s Livelihood and Striving to Resolve the People’s Urgent Concerns and Expectations,” provinces should integrate into national regional strategies. By leveraging tightly knit county-level medical consortia, they can compensate for shortages of high-quality resources through mechanisms for co-construction, resource sharing, and talent support.

### 4.5 Limitations and future research directions

This study has several limitations that should be acknowledged. First, the output indicators selected in this study are primarily measures of service volume. While such indicators capture aspects of process and quantity, they do not encompass outcome-oriented measures related to health status or service quality. This may compromise the comprehensiveness of the efficiency assessment. Consequently, this limitation affects the interpretation of the DEA results. For instance, a high DEA efficiency value merely indicates that a region efficiently produces service volume given its inputs; it cannot be extrapolated to infer superior service quality or health improvement outcomes. Second, the data envelopment analysis model employed in this study does not incorporate external environmental factors as control variables. While this approach facilitates the measurement of resource transformation capability from a purely technical perspective, it also implies that the estimated efficiency scores may be substantially influenced by prevailing environmental characteristics. Consequently, this may limit the explanatory power of the findings regarding causal relationships. Third, DEA results are sensitive to indicator selection and may not reflect stochastic variations. Finally, although we provide an optimization path by combining efficiency decomposition and redundancy analysis, due to the limitation of macro data and research methods, we fail to conduct detailed cost–benefit analysis, local adaptability assessment or priority ranking for each recommendation. Future research should incorporate quality-oriented indicators to complement existing measures of service volume. Furthermore, external environmental factors should be explicitly integrated into the analysis. This would provide a more robust explanation for regional efficiency disparities and strengthen the causal interpretation of the findings.

## Data Availability

The original contributions presented in the study are included in the article/Supplementary material, further inquiries can be directed to the corresponding author/s.
