# Supplementary figures and images for: Research on the spatial–temporal evolution of healthcare resource allocation efficiency in China
Source: Front Public Health. 2026 Jan 15;13:1729223. doi: 10.3389/fpubh.2025.1729223 (PMC12852340; doi:10.3389/fpubh.2025.1729223)

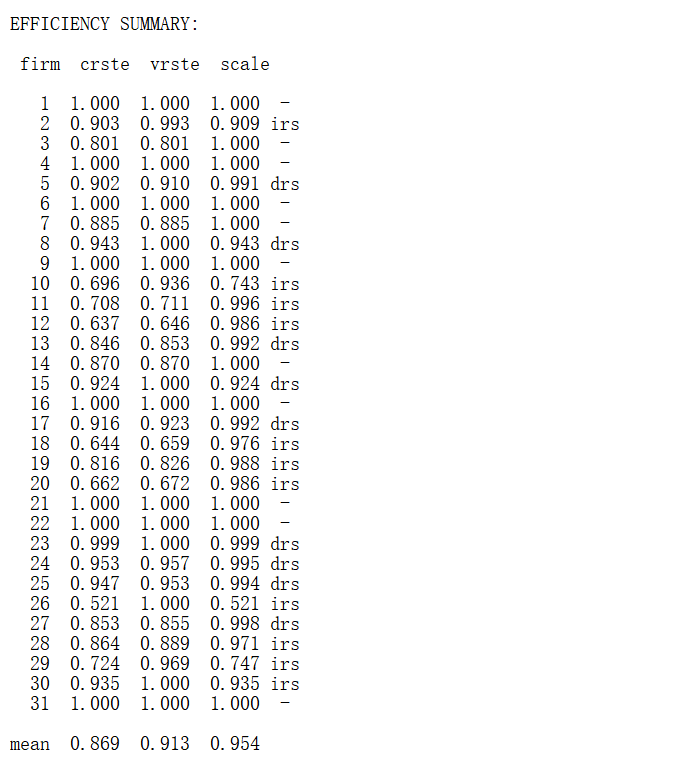

Supplement: Supplementary file 1 [file Data_Sheet_1.ZIP › Data Summary/Table 2/Specific values in Table 2.jpg]

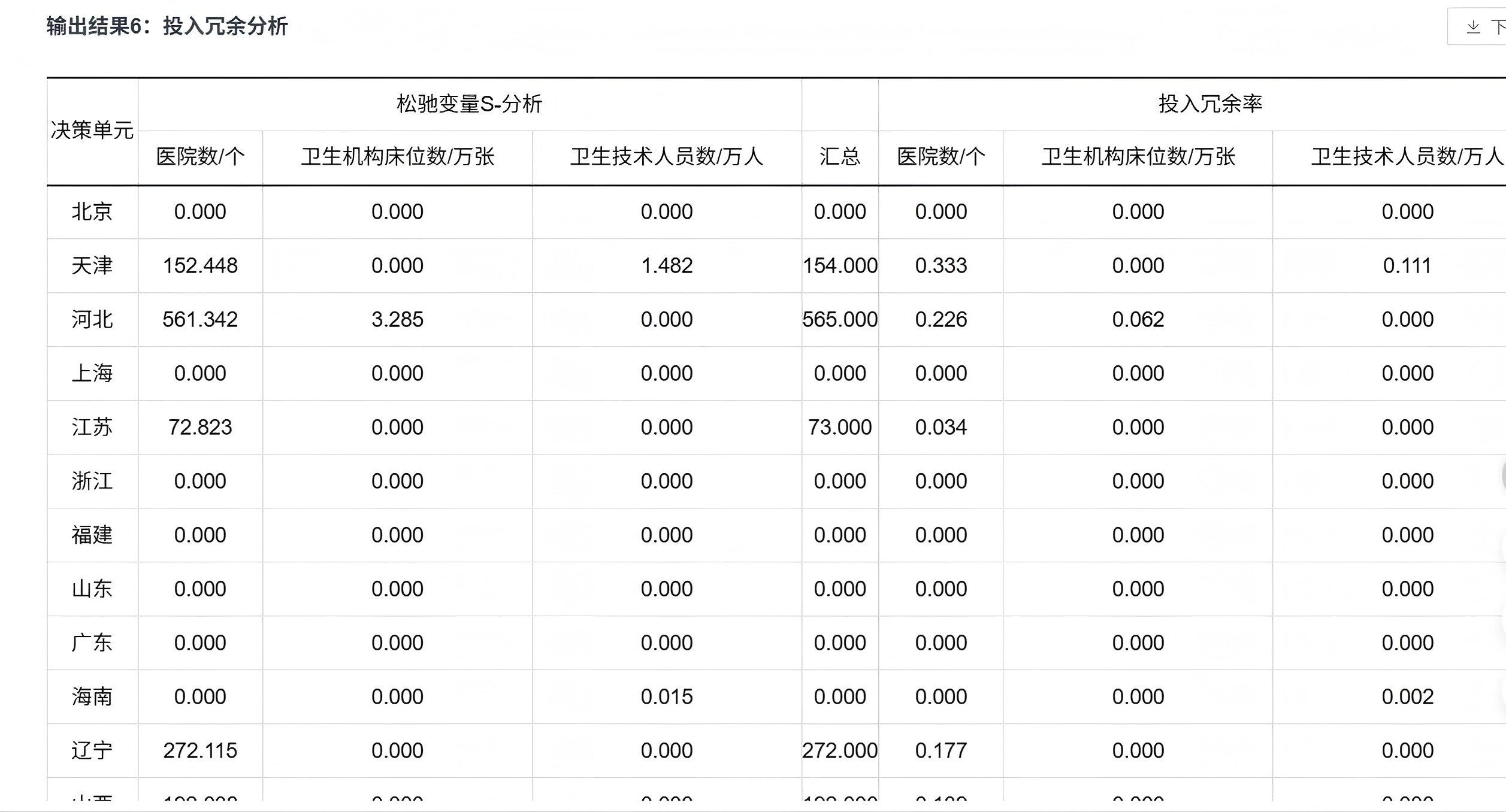

Supplement: Supplementary file 1 [file Data_Sheet_1.ZIP › Data Summary/Table 3 and Table 4/Table 3-Software analysis results.jpg]

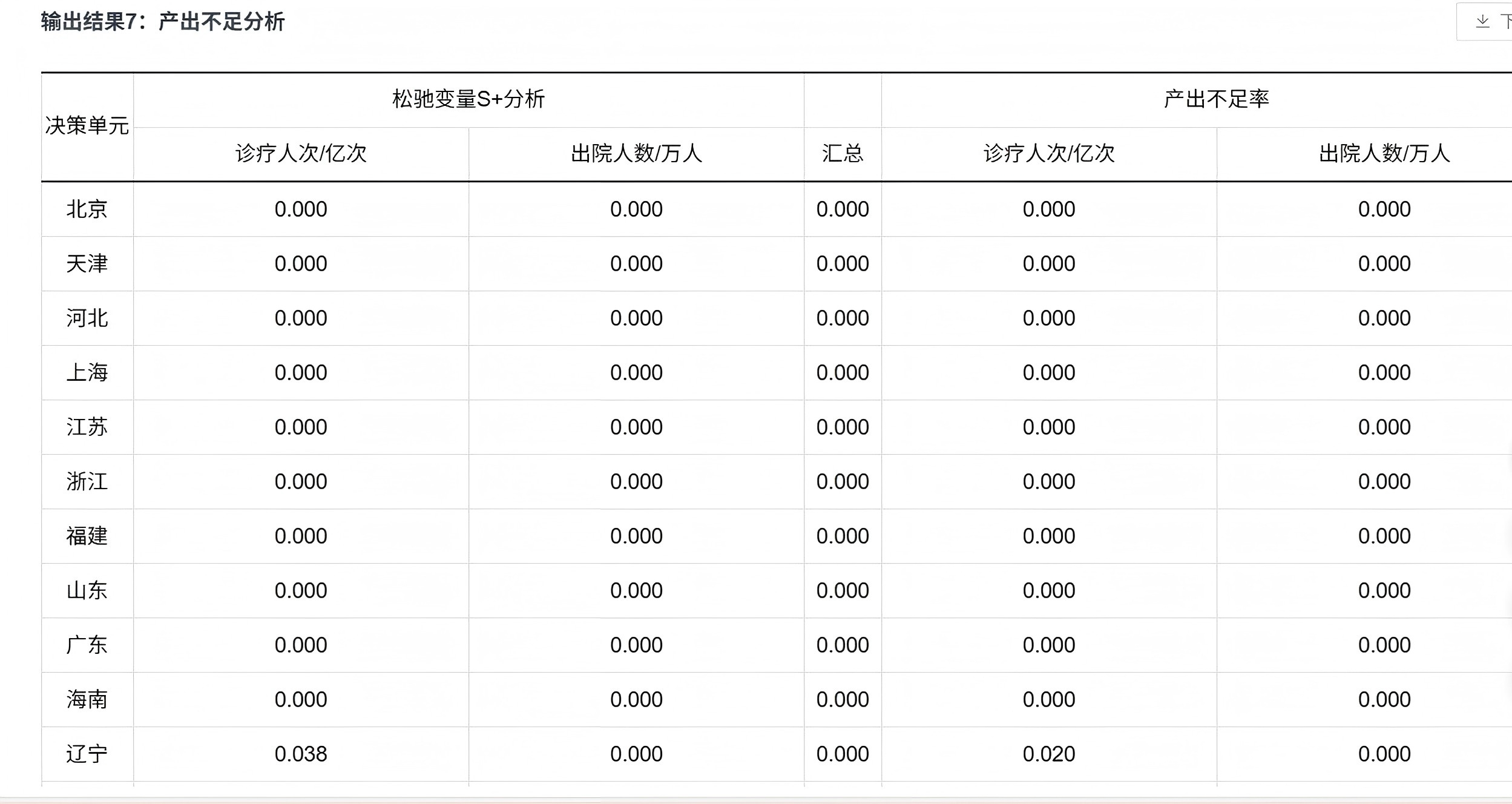

Supplement: Supplementary file 1 [file Data_Sheet_1.ZIP › Data Summary/Table 3 and Table 4/Table 4-Software analysis results.jpg]

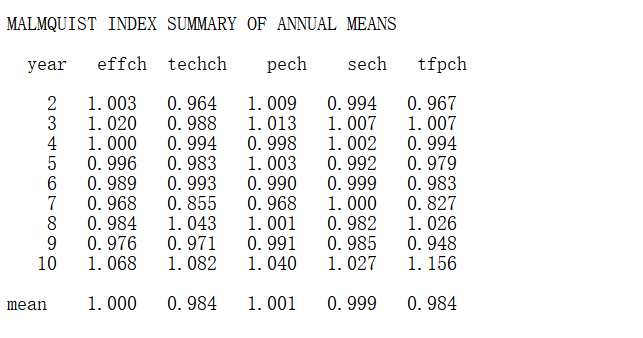

Supplement: Supplementary file 1 [file Data_Sheet_1.ZIP › Data Summary/Table 5 and Table 6/Table 5.jpg]

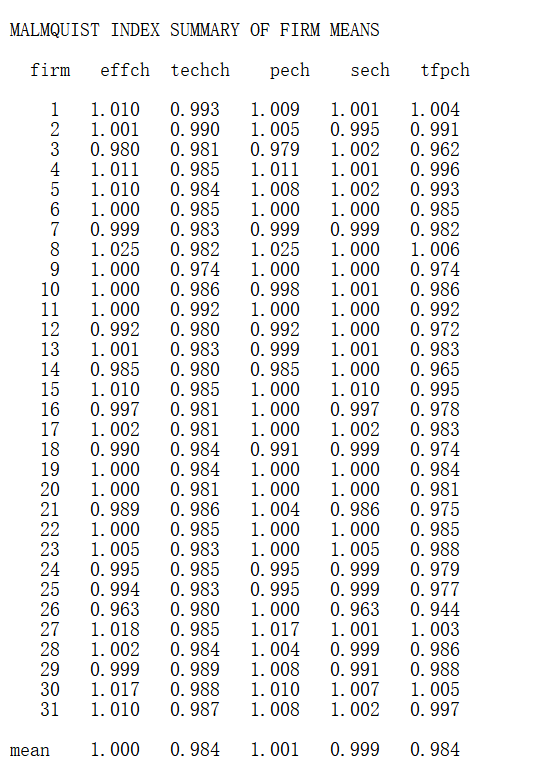

Supplement: Supplementary file 1 [file Data_Sheet_1.ZIP › Data Summary/Table 5 and Table 6/Table 6.jpg]
